# Identifying aging-related biomarkers in adipose tissue using integrative bioinformatics and machine-learning approaches: discovery of ELN, MXD1, and FGF21 as key genes

**DOI:** 10.3389/fendo.2025.1638343

**Published:** 2025-09-01

**Authors:** Xin Xie, Hongyan Wang, Fugang Xiao, Xiaoyan Jiang, Yan Chen, Chenghu Huang, Dongfeng Tang, Yanzhong Wang, Shunli Rui, Xi Cheng, Bo Deng, Gangyi Yang, Wuquan Deng

**Affiliations:** ^1^ Department of Endocrinology, the Second Affiliated Hospital of Chongqing Medical University, Chongqing, China; ^2^ Department of Endocrinology and Metabolism, Chongqing Emergency Medical Center, Chongqing University Central Hospital, School of Medicine, Chongqing University, Chongqing, China; ^3^ Department of Endocrinology, Bishan Hospital of Chongqing, Bishan Hospital of Chongqing Medical University, Bishan, Chongqing, China; ^4^ School of Life Course and Population Sciences, King’s College London, London, United Kingdom

**Keywords:** aging, biomarker, adipose tissue, bioinformatics, machine learning

## Abstract

**Background:**

Adipose tissue plays a critical role in aging and age-related diseases. However, the specific molecular and cellular alterations associated with aging in adipose tissue remain incompletely understood.

**Methods:**

Aging-related differentially expressed genes (DEARGs) were identified by intersecting differentially expressed genes (DEGs) in adipose tissue, age-related genes (ARGs), and human genes linked to aging. Functional enrichment analysis was conducted to explore the potential roles of these DEARGs. Protein-protein interaction (PPI) networks were analyzed using STRING, and hub DEARGs were identified via least absolute shrinkage and selection operator (LASSO) analysis. Oil Red O staining was used to confirm adi-pocyte differentiation, and D-galactose treatment induced cellular senescence. Validation of hub DEARG expression was conducted in an independent dataset and confirmed using quantitative polymerase chain reaction (qPCR) both *in vitro* and *in vivo*.

**Results:**

Forty-nine DEARGs were identified, with functional enrichment analyses revealing significant roles in glucose homeostasis and key aging pathways, including the FoxO and JAK-STAT signaling pathways, Th17 cell dif-ferentiation, growth hormone signaling, the adiponectin pathway, and AMPK pathway. Five hub genes (PCK1, ELN, MXD1, STAT3, and FGF21) were selected through interaction network anal-ysis and LASSO regression. Expression levels of three DEARGs (ELN, MXD1, and FGF21) were validated by qPCR and an independent dataset.

**Conclusions:**

This study identified three DEARGs (ELN, MXD1, and FGF21) as potential biomarkers of adipose tissue aging, suggesting their role in organismal aging and age-related disease pathways.

## 1 Introduction

Lipids play vital roles in living organisms, serving not only as key components of cell membranes but also in energy storage and signaling pathways that regulate cellular functions. Disruptions in lipid metabolism have been implicated in numerous diseases, including atherosclerosis, cancer, non-alcoholic steatohepatitis, and chronic kidney disease (1–4). Beyond disease, lipid metabolism has also been identified as a crucial regulator of aging and lifespan, with alterations in lipid pathways contributing to the aging process (5, 6).

The exact mechanisms by which lipid composition and metabolism change with aging—and whether modulating these changes can extend lifespan—remain areas of active investigation.

Among various tissues, adipose tissue has garnered particular interest due to its distinct and complex responses to age-related changes. Studies have shown that adipose tissue is not merely a passive energy reservoir; rather, it plays an active role in systemic aging. For example, age-associated activation of immune cells, a hallmark of aging, is especially pronounced in white adipose tissue and can be detected early in the aging process. Progenitor cells within adipose tissue also display heightened sensitivity to the aging microenvironment, as demonstrated by parabiosis experiments, highlighting adipose tissue’s potential role in modulating age-related physiological changes (7).

Adipose tissue may mediate systemic aging effects through several mechanisms. Adipocytes, as potent endocrine cells, secrete numerous bioactive peptides, known as adipokines, along with extracellular vesicles that influence nearby tissues and distant organs (8, 9). These secreted factors can affect metabolic and inflammatory responses, thereby impacting aging and longevity. Recent studies have identified diverse adipocyte subtypes with distinct secretomes, suggesting that the effects of adipose tissue on aging may vary across different fat depots and influence local tissues in unique ways (10–12). Furthermore, adipose tissue serves as a niche for pluripotent progenitor cells that support tissue development and regeneration, potentially affecting tissue homeostasis throughout life (13, 14).

Despite the known significance of adipose tissue in aging, a systematic profiling of the molecular changes associated with adipose tissue aging remains limited. To address this gap, our study utilized adipose tissue-specific gene expression profiles, integrating findings from aging-related databases with bioinformatics analyses and experimental validation, to identify biomarkers linked to adipose tissue senescence. By identifying these biomarkers, we aim to enhance understanding of adipose tissue aging and provide insights that may inform future therapeutic strategies targeting age-associated diseases.

## 2 Results

### 2.1 Identification and functional enrichment of DEARGs for adipose tissue aging

**Figure 1:**
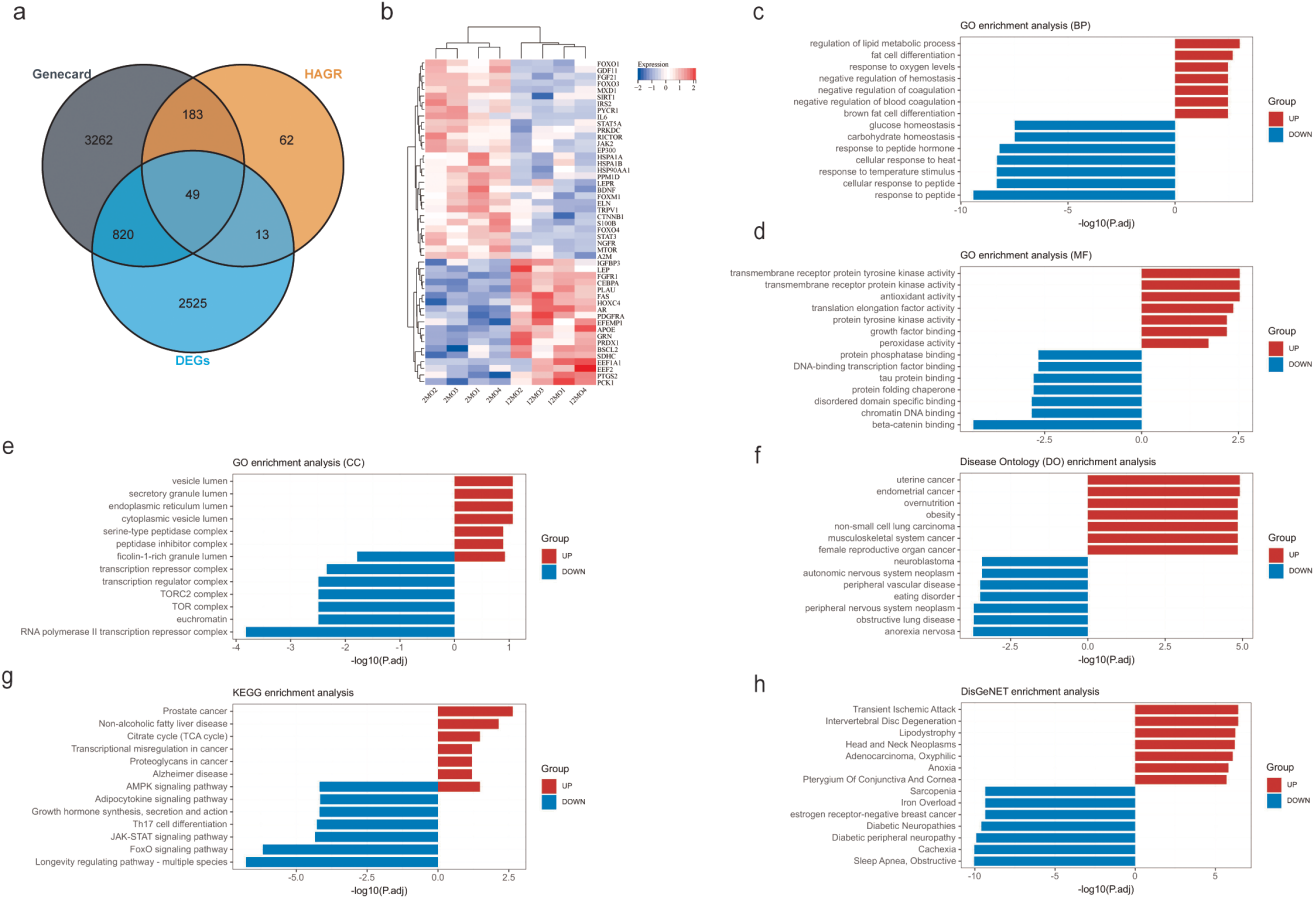
Discovering and enhancing the roles of differentially expressed adipocyte-related genes in the aging process: **(a)** Using a Venn diagram to identify DEARGs; **(b)** Heat map showing differences in DEARGs between the young and aged mice; **(c)** GO biological processes analysis for DEARGs; **(d)** Performing GO molecular function analysis on differentially expressed autophagy-related genes; **(e)** GO cellular component analysis for DEARGs; **(f)** Disease Ontology (DO) enrichment analysis for DEARGs; **(g)** Performing KEGG pathway analysis for DEARGs; **(h)** Conducting DisGeNET enrichment analysis for DEARGs.

A total of 49 DEARGs were identified by comparing DEGs in adipose tissue, ARGs, and human genes associated with aging (**Figure 1a**). The expression patterns of these 49 DEARGs in 12-month-old and 2-month-old mice were visualized using a heatmap (**Figure 1b**). Functional enrichment analysis was performed on these DEARGs to explore their biological roles (**Supplementary Table S1**).

For biological processes (BP), significant overlaps were found in glucose homeostasis, response to peptide hormones, thermal stimulus response, and peptide response (**Figure 1c**). In terms of molecular functions (MF), notable overlaps were observed in beta-catenin binding, DNA-chromatin binding, binding to unfolded protein domains, and protein chaperone folding (**Figure 1d**). Cellular components (CC) revealed overlap in the RNA polymerase II transcription repressor complex, euchromatin, TOR complex, and transcription regulatory complexes (**Figure 1e**). Disease Ontology (DO) analysis indicated overlaps with endometrial cancer, endometrioid carcinoma, hyperalimentation, and obesity (**Figure 1f**). KEGG pathway analysis suggested that pathways such as the longevity-regulating pathway, FoxO signaling pathway, JAK-STAT signaling pathway, Th17 cell differentiation, growth hormone synthesis/secretion/action, adiponectin signaling pathway, and AMPK signaling pathway may be involved in adipocyte changes during aging (**Figure 1g**). DisGeNET enrichment analysis indicated overlaps with sleep apnea, cachexia, diabetic peripheral neuropathy, diabetic neuropathies, estrogen receptor-negative breast cancer, iron overload, and sarcopenia (**Figure 1h**).

### 2.2 Identification of hub DEARGs with the LASSO algorithm

**Figure 2:**
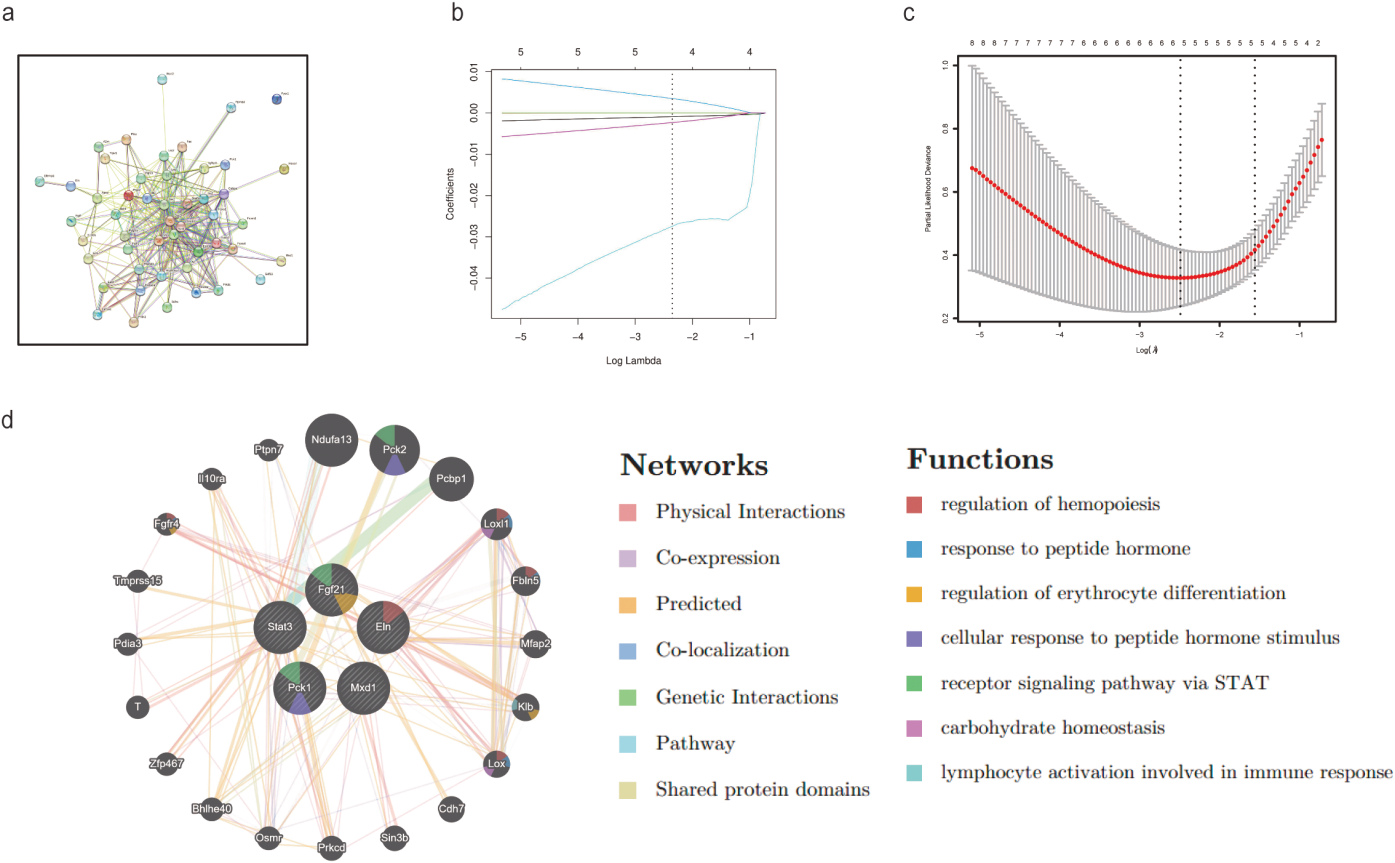
Discovering key DEARGs associated with the aging process of adipocytes: **(a)** PPI network of DEARGs; **(b, c)** Based on the LASSO regression model, five genes with non-zero coefficients were chosen using the optimal lambda; **(d)** The GeneMANIA database displays DEGs and the networks of genes they co-express with.

To identify hub DEARGs involved in adipose tissue aging, the 49 DEARGs were analyzed using the STRING database to construct a protein-protein interaction (PPI) network with 49 nodes and 295 edges (**Figure 2a**, **Supplementary Table S2**). Feature selection using LASSO regression identified five hub DEARGs—PCK1, ELN, MXD1, STAT3, and FGF21—which showed significant non-zero regression coefficients with an optimal lambda value of 0.095 (**Figures 2b, c**).

Further co-expression analysis was performed using the GeneMANIA database, revealing an intricate PPI network with various interaction types, including 1.04% common protein domains, 1.57% colocalization, 1.81% genetic interactions, 2.07% pathways, 8.09% additional elements, 17.96% co-expression, 22.45% anticipated interactions, and 45.00% direct interactions (**Figure 2d**). These genes were found to play key roles in energy metabolism, extracellular structure organization, fibroblast growth factor responses, short-chain fatty acid metabolism, protein oxidation, growth factor binding, and triglyceride metabolism, suggesting their significant involvement in the aging of adipose tissue.

### 2.3 Datasets and qPCR Validation of hub DEARGs for adipose tissue aging

**Figure 3:**
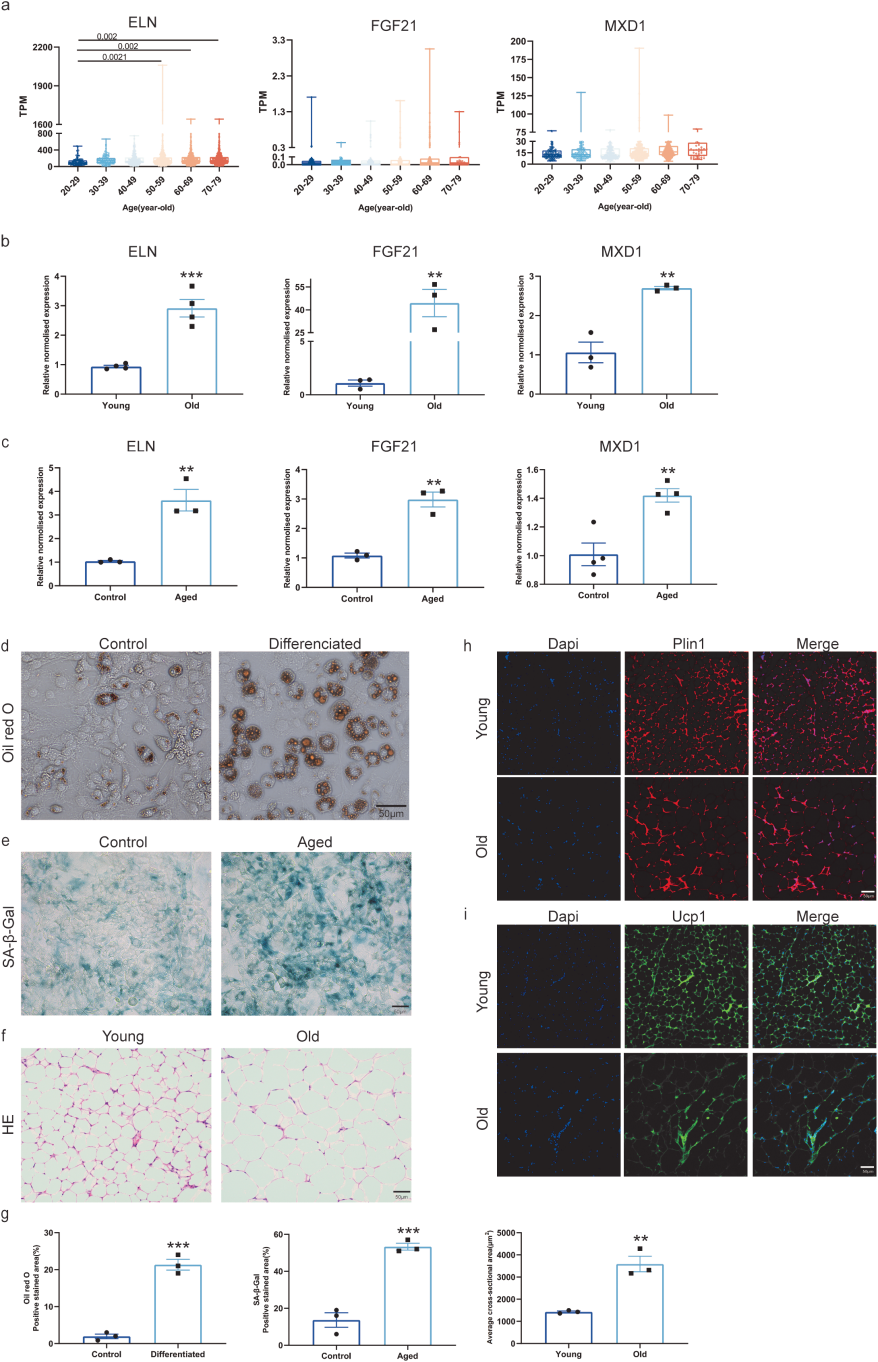
Validation of hub DEARGs using qPCR and datasets: **(a)** Validation of hub DEARGs dataset through the Integration Platform of ADEIP; **(b)** Validation of central DEARGs in white adipose tissue from young and old mice using qPCR; **(c)** qPCR validation of hub DEARGs in adipocyte; **(d)** Representative images of Oil red O staining of adipocyte. Scale bar 50 μm; **(e)** SA-β-gal staining of adipocytes. Scale bar 50 μm; **(f)** Representative H&E stained images of white adipose tissue sections from young and old mice (20x magnification, scale bars 50 μm) (Images representative of 3 separate experiments); **(g)** Quantification of **(d–f)**; **(h, i)** Immunofluorescence examination of PLIN1 (red), UCP1 (green), and DAPI (blue) in WAT from young and old mice. Scale bar 50 μm. Mean values ± SEM are provided in the data presentation. Analysis was conducted using a two-tailed Student’s t-test. ** means p<0.01, *** means p<0.001.

To validate the expression of the identified hub DEARGs—PCK1, ELN, MXD1, STAT3, and FGF21—in human adipose tissue, data from the Integrating Platform of ADEIP were analyzed. The results showed that ELN, MXD1, and FGF21 exhibited upregulated expression in the adipose tissue of older individuals (**Figure 3a**).

*In vitro*, differentiated adipocytes were confirmed by Oil Red O staining (**Figure 3d**). To establish an *in vitro* model of cellular aging, adipocytes were treated with D-galactose (**Figure 3e**). H&E staining (**Figure 3h**) and immunofluorescence (**Figure 3i**) of white adipose tissue (WAT) from young and old mice further supported these findings.

The expression of the hub DEARGs was also validated by qPCR in the D-galactose-induced aging model and in WAT isolated from 2-month-old and 12-month-old mice. The mRNA levels of ELN, MXD1, and FGF21 were significantly higher in older adipocytes compared to younger controls (**Figure 3b**). This validation confirmed that ELN, MXD1, and FGF21 are upregulated during adipose tissue aging, supporting their potential as biomarkers for aging in adipose tissue (**Figure 3c**).

## 3 Discussion

Cellular senescence was initially described as the gradual decline in the replicative ability of primary fibroblasts *in vitro*, leading to irreversible cell cycle arrest (15). Replicative senescence is triggered by telomere shortening, a form of DNA damage that can also result from oxidative stress, radiotherapy, chemotherapy, and oncogenic signals (16, 17). During *in vitro* studies, DNA damage-induced mitotic arrest initiates a secretory response characterized by the release of numerous cytokines, collectively known as the senescence-associated secretory phenotype (SASP).

A robust link between energy balance and aging has been observed across diverse organisms, from single-celled eukaryotes to humans. Cellular lipid metabolism, largely driven by mitochondrial β-oxidation, is a primary energy source that also generates oxidative stress and reduces glucose uptake (18, 19). Disruptions in β-oxidation can result in the accumulation of toxic byproducts, contributing to cellular senescence. Adipose tissue, integral to lipid distribution and storage, plays a crucial role in energy homeostasis, cellular stress response, and aging across species. Single-cell transcriptomic studies have indicated that adipose tissue may be one of the first organs to exhibit age-related changes, potentially influencing the aging of other tissues. This theory is supported by the universal presence of age-related shifts in adipose tissue composition—such as the loss of subcutaneous white adipose tissue (sWAT) and increased fat in bone marrow—across species.

sWAT, located beneath the skin’s dermis, specializes in energy storage and release as triglycerides and fatty acids via lipid droplets within white adipocytes. These lipid droplets can occupy over 90% of cellular volume, effectively isolating stored lipids. During fasting, triglycerides are hydrolyzed to release fatty acids into the bloodstream, fueling systemic energy needs (19). The lipid storage function of sWAT is facilitated by large lipid droplets; however, dysfunction in sWAT can lead to ectopic fat deposition, resulting in conditions such as lipotoxicity and insulin resistance (20).

Aging primarily affects white adipose tissue (WAT) by promoting hypertrophy in intra-abdominal fat and reducing sWAT, which diminishes its lipid storage capacity (21). While hypertrophy increases lipid storage, larger adipocytes are more insulin-resistant and exhibit higher basal lipolysis (22). This compromised lipid accumulation function in sWAT increases the metabolic burden on other tissues, disrupting redox balance and contributing to cellular senescence.

In this study, we identified 49 DEARGs by overlapping DEGs in adipose tissue with known aging-related genes (ARGs) and human aging genes. Functional KEGG enrichment analysis suggested these DEARGs may regulate aging pathways, including FoxO signaling, JAK-STAT signaling, Th17 cell differentiation, growth hormone synthesis/secretion, adiponectin signaling, and AMPK signaling. Thus, these DEARGs likely play a role in adipose tissue aging.

To further narrow down DEARGs of particular relevance to adipose tissue, we applied the LASSO algorithm, which identified five hub genes: PCK1, ELN, MXD1, STAT3, and FGF21. Subsequent validation through ADEIP and qPCR confirmed that ELN, MXD1, and FGF21 were associated with age-related changes in adipose tissue. Each of these genes has been previously implicated in aging processes, supporting their potential role as markers for adipose tissue aging.

ELN (encoding tropoelastin) is essential for elastic fiber production, which supports tissue elasticity. Age-related declines in elastic fibers and the accumulation of bioactive elastokines, products of elastin degradation, are linked to aging. Reduced ELN expression may lead to premature tissue aging and structural deterioration, affecting tissue functionality and contributing to various connective tissue disorders (23–25).

MXD1 (also known as Mad1) is a transcriptional repressor involved in regulating cellular processes such as transformation, differentiation, proliferation, and apoptosis (26–28). Reduced MXD1 levels have been associated with increased cell survival and invasiveness in cancers, including pancreatic, breast, and gastric cancers (29–31). Conversely, elevated MXD1 levels can inhibit cancer cell growth (32, 33), further highlighting its role in cell cycle regulation and potential relevance to aging.

Circulating FGF21 is a hormone whose circulating levels increase with age in both rodents and humans (34–36). Studies in animal models have shown that elevated FGF21 levels are associated with longevity. FGF21 mitigates inflammation by promoting the M1-to-M2 macrophage transition, thereby protecting tissues from age-related damage, such as liver fibrosis and chronic kidney disease associated with obesity (37–39). Recognized for its longevity-promoting and anti-inflammatory effects (37, 40–43), FGF21 emerges as a critical regulator of aging processes.

By overexpressing or knocking down ELN, MXD1 or FGF21 at the cellular level respectively, and detecting the expression of key molecules in senescence-related signaling pathways such as FoxO and JAK-STAT, the specific mechanisms of these genes in the process of cellular senescence were further revealed.

## 4 Limitations and future directions

While our study provided insights into DEARGs associated with adipose tissue aging, it has limitations. Specifically, we used D-gal-induced aging in cultured cells and WAT from aged mice rather than clinical samples. Although this approach allowed for controlled investigation, it may not fully replicate *in vivo* conditions. Although the current model (C57BL/6 mice) can simulate a specific state of aging, it cannot reproduce the three core characteristics of human aging: decades of accumulated environmental exposure (chronic low inflammation and epigenetic drift), multi-system network decline (imbalance of neuro-endocrine-immune axis), and biological effects of psychosocial factors (chronic stress accelerates telomere shortening). In future research, we will design a stratified clinical translation pathway: Phase 1: Cross-sectional biomarker validation (0–2 years). Study population: Community-dwelling naturally aging populations, centenarians, and their direct descendants. Core measures: Multimodal omics profiling and clinical functional assessments (weakened index, cognitive scales). Phase 2: Longitudinal intervention study (3–5 years). From Phase 1, we will identify high-risk biomarker populations and conduct randomized double-blind trials based on the target molecules identified in this study, with the primary endpoint being biomarker improvement rates. While the models presented in this paper provide a controlled platform for mechanism exploration, aging fundamentally represents a systemic collapse of biological systems across temporal and spatial dimensions. Future research must anchor laboratory findings to human physiological networks through deep phenotyping analysis, with particular focus on three key areas: inter-organ aging signal transmission (e.g., the muscle-brain axis), microbiome-host co-aging dynamics, and the biological embedding mechanisms of social determinants (SDoH). To strengthen our findings, future studies will include clinical tissue samples, providing direct relevance to human aging and potentially broadening the applicability of our results. Current cross-sectional study designs can only identify potential biomarkers at a single time point in aged mice. Longitudinal studies are crucial for revealing the expression changes of ELN, MXD1, and FGF21 during aging, which also serve as key evidence for evaluating these biomarkers’ predictive potential in age-related diseases. We will conduct longitudinal studies as a critical follow-up step to further expand the research value of these preliminary findings. This approach aims to enhance the robustness and translational potential of our findings.

## 5 Conclusions

In summary, this study employed bioinformatics analysis to identify three key genes—ELN, MXD1, and FGF21—associated with the aging process in adipose tissue. These genes are likely to play a central role in organismal aging and could contribute to the development of age-related diseases. Through this analysis, we provide valuable insights that enhance our understanding of adipose tissue aging and offer potential targets for interventions aimed at mitigating aging-related declines in adipose tissue function. Future research based on these findings may further clarify the roles of these genes in age-associated pathologies and promote advancements in therapies targeting cellular aging.

## 6 Materials and methods

**Figure 4:**
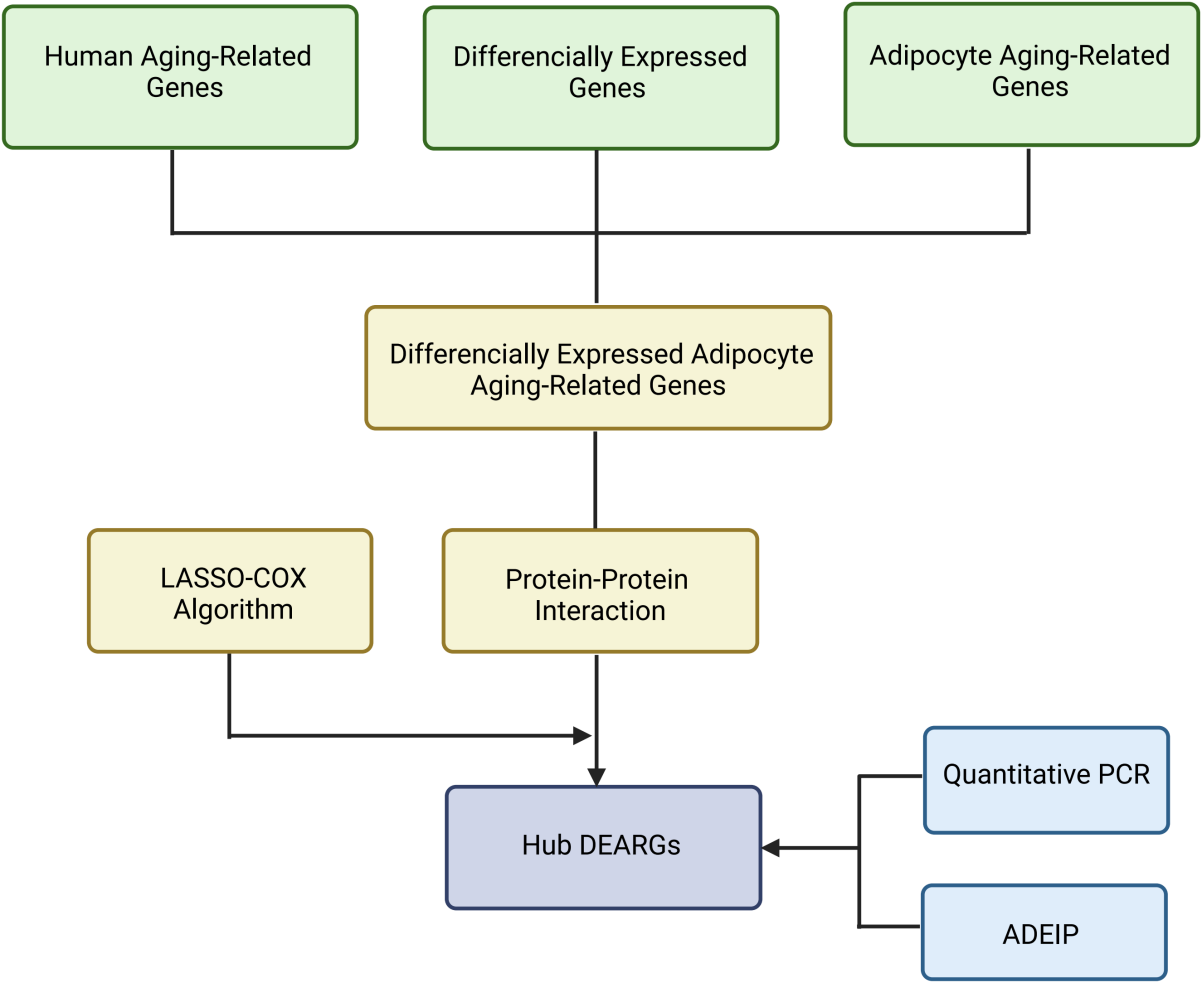
The flowchart of adipocyte aging research using comprehensive bioinformatics methodsCreated with BioRender.com.

This study aimed to identify gene sets associated with adipose tissue aging, focusing on age-related genes (ARGs) to uncover potential biomarkers. The overall study workflow is illustrated in **Figure 4**.

### 6.1 Data collection and preprocessing for DEGs and ARGs identification in adipose tissue

To explore aging-associated genes in adipose tissue, we sourced 4,314 ARGs from the GeneCards database, with each gene having a relevance score greater than 5 (**Supplementary Table S3**). Additionally, we obtained a set of 307 genes specifically associated with human aging from the Human Aging Genomic Resources (HAGR) database (https://genomics.senescence.info/; see (**Supplementary Table S4**).

We retrieved the CRA015560 mRNA expression profiling dataset from the Genome Sequence Archive (GSA) (https://ngdc.cncb.ac.cn/gsa/) to analyze differentially expressed genes (DEGs) between young and aged mice. Comparative analysis was performed using the limma R package to identify significant gene expression differences between the youthful and aged cohort. Genes were considered differentially expressed if they met the criteria of |fold-change (FC)| > 1.5 and p-value < 0.05.

### 6.2 Functional enrichment analysis of DEGs and DEARGs

To investigate the biological significance of DEGs and differentially expressed age-related genes (DEARGs) in adipose tissue aging, functional enrichment analysis was conducted. Gene annotations were sourced from the Human Phenotype Ontology (HPO), Gene Ontology (GO), and Kyoto Encyclopedia of Genes and Genomes (KEGG) within the Molecular Signatures Database (MSigDB) and DAVID databases. Enrichment analyses for DEGs were performed using the R package ClusterProfiler alongside DAVID. Functional categories and pathways with a p-value < 0.05 were considered statistically significant.

### 6.3 Identification of central biomarkers using protein-protein interaction analysis

To identify key biomarkers within DEARGs, we performed a PPI analysis using the STRING database, applying a confidence score threshold of >0.4. The interaction network was visualized in Cytoscape 3.8.1. We used the MCODE plugin within Cytoscape to extract major functional modules, with selection parameters set to K-Core = 2, Cut Grade = 2, Maximum Depth = 100, and Cut Node Score = 0.3. Each gene node was evaluated using the Maximum Clique Centrality (MCC) score within the cytoHubba plugin of Cytoscape, and the top 10 genes with the highest MCC scores were identified as key nodes. Further refinement of important biomarkers was achieved through Cox regression analysis with the least absolute shrinkage and selection operator (LASSO) for gene selection. The optimal penalty parameter was chosen using triple cross-validation to minimize cross-validation error, as implemented in the ‘glmnet’ R package. Additionally, we utilized GeneMANIA (http://genemania.org/; last accessed on May 1, 2023) to validate PPI networks of key biomarkers.

### 6.4 Validation of hub biomarkers using age-dependent expression and immune profiles across human tissues

We employed the Age-Dependent Expression and Immune Profiles (ADEIP) database to validate hub biomarkers. ADEIP provides gene expression and cell proportion data across different age groups and genders (http://gb.whu.edu.cn/ADEIP/). Using this resource, we examined the expression profiles of identified biomarkers across age groups to confirm their relevance to aging.

### 6.5 Animal models

Animal experiments were conducted in accordance with the ethical guidelines set by the Chongqing University Animal Experiment Ethics Committee. C57BL/6 mice were obtained from Gempharmatech Co., Ltd (Guangzhou, China). Mice were housed in a temperature-controlled facility with a 12-hour light-dark cycle and provided ad libitum access to food and water. Male mice of specific ages were used for all experiments, with at least three animals per group in each experiment. Mice were anesthetized by continuous inhalation of isoflurane (3%). Euthanasia was performed by exsanguination under anesthesia, followed by cervical dislocation as a secondary euthanasia procedure.

### 6.6 Cell culture and treatment

The 3T3-L1 preadipocyte cell line was sourced from the Stem Cell Bank of the Chinese Academy of Sciences. Cells were cultured in Dulbecco’s Modified Eagle Medium (DMEM, Vivacell) supplemented with 10% newborn calf serum (CS, Adamas Life, C8231). Cells were grown until confluence, at which point adipogenesis was induced. The differentiation process began with pre-culturing cells in basic medium for 2 days, followed by treatment with a differentiation medium containing 10 µg/mL insulin, 0.25 µM dexamethasone, and 500 µM IBMX for 2 days. Subsequently, cells were cultured in a growth medium containing only insulin for an additional 2 days. Fully differentiated adipocytes were cultured in DMEM supplemented with 10% fetal bovine serum (FBS, Vivacell), with the medium changed every 2 days until adipogenesis was complete. Differentiated adipocytes were identified by their characteristic morphology of multivesicular lipid droplets, which stained red with Oil Red O solution. To induce cellular senescence, adipocytes were exposed to 40 g/L D-(+)-galactose (Beyotime, ST1218) for 48 hours.

### 6.7 Senescence-associated β-galactosidase activity

SA-β-gal activity was assessed using a senescence β-galactosidase staining kit (C0602, Beyotime, Shanghai, China), following the manufacturer’s instructions. The percentage of SA-β-gal-positive cells was quantified to assess the extent of cellular senescence.

### 6.8 Oil red O staining

Oil Red O staining was performed using a modified Oil Red O staining kit (Beyotime, C0158), according to the manufacturer’s instructions. After staining, cells were subjected to microscopic analysis to assess lipid accumulation.

### 6.9 RNA isolation and qPCR

Total RNA was extracted from mouse white adipose tissue (WAT) and cultured cells. For tissue RNA extraction, approximately 50 mg of WAT was placed in tissue grinding tubes with zirconium beads and 500 μL TRIzol reagent (Invitrogen, 15596026CN). Tissues were homogenized using a Servicebio™ homogenizer with two 30-second pulses at 6 m/s, separated by a 15-second pause. For cell RNA isolation, the NcmSpin Cell/Tissue Total RNA Kit (NCM Biotech, M5105) was used. RNA concentration and quality were assessed with a Thermo Scientific™ NanoDrop™ One spectrophotometer. One microgram of RNA was reverse transcribed into complementary DNA (cDNA) using the ABScript Neo RT Master Mix (ABclonal, RK20433), along with the gDNA Remover Kit. Quantitative PCR (qPCR) was performed using a Bio-Rad™ CFX96 Touch Real-Time PCR Detection System with 2X Universal SYBR Green Fast qPCR Mix (ABclonal, RK21203) and gene-specific primers (**Supplementary Table S5**). The ΔΔ-CT method was applied to analyze gene expression, with Actin as the internal control.

### 6.10 Histological analysis

Slides were prepared according to a previous report (44).

Hematoxylin and Eosin (H&E) Staining: Tissue sections were rehydrated through a graded alcohol series (xylene for 3 minutes, 100% alcohol for 1 minute twice, 95% alcohol for 1 minute twice, and water for 1 minute), followed by staining with H&E. The slides were dehydrated in reverse order, mounted with Cytoseal 60, and analyzed using an Olympus VS200 inverted microscope.

Immunofluorescence Staining: Slides were rehydrated according to the following protocol: xylene (20 min x 3 times); 100% reagent alcohol (5 min x 2 times); 95% reagent alcohol (5 min x 1 time); 85% reagent alcohol (5 min x 1 time); 75% reagent alcohol (5 min x 1 time); 50% reagent alcohol (5 min x 1 time); PBS (5 min x 1 time). Sodium citrate solution (BOSTER, AR0024) was utilized for antigen retrieval. The slides were rinsed three times with 1x PBS. Slides were incubated with the primary antibody in 1X TBS containing 5% goat serum at 4°C overnight after being blocked for 30 minutes with 5% goat serum in 1X TBS. Primary antibodies included Rabbit anti-Perilipin A (1/200; HUABIO ET1703-38) and Mouse anti-Ucp1 (1/200; Santa Cruz sc-518024). Invitrogen secondary antibodies were diluted to 1/500 and then incubated for 1 hour at room temperature. The following secondary antibodies were used: 555 Goat anti-Rabbit or 488 Goat anti-Mouse. The slides were cleaned and dyed using DAPI (Servicebio G1012) for 10 minutes, followed by application of mounting solution (Solarbio S2100). Fluorescent images were recorded using the Olympus VS200 inverted microscopy system.

### 6.11 Quantification and statistical analysis

Statistical significance was assessed using a two-tailed Student’s t-test to compare two groups, and one-way ANOVA was used for multiple group comparisons. Data are expressed as means ± SEM. P-values less than 0.05 were considered statistically significant. For microscopy images, three randomly selected fields from at least three mice per cohort were analyzed using NIH Fiji ImageJ software. H&E, Oil Red O, SA-β-gal, and immunofluorescence images were captured from a minimum of three to four replicates per group. RNA-seq data were analyzed for statistical significance, heat map generation, and KEGG pathway analysis using RStudio. Graphical representation and statistical analysis were performed using GraphPad Prism 7-9, and initial data collection was performed using Microsoft Excel.

## Data Availability

The original contributions presented in the study are included in the article/**Supplementary Material**. Further inquiries can be directed to the corresponding authors.
